# Supplementary material for: Structure and Function of the ABCD1 Variant Database: 20 Years, 940 Pathogenic Variants, and 3400 Cases of Adrenoleukodystrophy
Source: Cells. 2022 Jan 14;11(2):283. doi: 10.3390/cells11020283 (PMC8773697; doi:10.3390/cells11020283)
Supplement: Supplementary file 1 [file cells-11-00283-s001.zip › cells-1491702-supplementary.pdf]

Supplemental Figure S1 to Structure and function of the ABCD1 variant database: 20 years, 940 pathogenic variants and 3400 cases of adrenoleukodystrophy

CLUSTAL Omega (1.2.4) multiple sequence alignment of ABCD1 proteins from 54 different species

```
Human                   mpvlsrprpwrgntlkrtavllalaaygahkvyplvrqclaparglqapageptqeasgv  60
Chimpanzee              mpvlsrprpwrgntlkrtavllalaaygahkvyplvrqclaparglqapageptqeasgv  60
Elephant                mpvlstprpsrvttlkrtavvlaltaygahkiyplvrqclgpargppgpagepaqeasga  60
Gorilla                 mpvlsrprpwrgntlkrtavllalaaygahkvyplvrqclaparglqapageptqeasgv  60
Green-Monkey            -mpvprprpwrgstlkrtavllalaaygahkvyplvrqclapargsqapakestqeasg-  58
Jerboa                  mpvlstprssrvttlkrtavvlaltaygvhkiyplvrqcltpargpqvpageptqeapga  60
Macaque                 mpvlsrprpwrgstlkrtavllalaaygvhkvyplvrqclapvrgsqapareptqessga  60
Marmoset                mpvlsmprpwrgstlkrtavllalaaygahkayplvrqclavargpqapardptqeasga  60
Mouse                   mpvlstprpsrvttlkrtavvlaltaygvhkiyplvrqcltpargpqvpageptqeasga  60
Orangutan               mpvlsrprpwrgstlkrtavllalaaygahkvyplvrqclasargpqapageptqeasgv  60
Panda                   mpvlstprssrvttlkrtalvlaltaygahkiyplvrqylaatrgpqvpageatqeisga  60
Pig                     mtvlstprpsrvttlkrtavvlaltaygahkiyplvrqclapargpqgpvrelaqevpga  60
Pika                    mpvlsttrpsrattlkrtavimalaaygahklyplvrqclglargpqvprgeptqeapgs  60
Prairie-deer-mouse      mpvlstprssrvttlkrtavvlaltaygvhkiyplvrqcltpargpqvptgepmqetsga  60
Rabbit                  mpvlstarpsrvttlkrtavvvaltaygvhkiyplvrqclapargpqvprgeptqeasga  60
Rat                     mpvlstprpsrvttlkrtavvlaltaygvhkiyplvrqcltpargpqvpagestqeasga  60
Rhesus-Monkey           mpvlsrprpwrgstlkrtavllalaaygvhkvyplvrqclapargsqapareptqessga  60
Seal                    mpvlstsrssrvttlkrtalvlaltaygahkiyplvrqylaatrgpqvpagestqevsga  60
Squirrel-Monkey         mpvlstprpwrgstlkrtavllalaaygahkayplvrqclalargpqaptrdptqeasga  60
Tarsier                 mpvlstprpsrvttlkrtavvlalaaygvhkiyplvrqclapargppglareptqeapga  60
Tasmanian-devil         ------------------------------------------------------------  0
Walrus                  mpvlstprssrvttlkrtalvlaltaygahkiyplvrqylaatrgpqvpagespqevsga  60
Water-buffalo           ------------------------------------------------------------  0
White-Rhinoceros        mpvlsiprpsrvttlkrtavllaltaygahkiyplvrqclvpargpqvpagestqetsga  60
Yak                     mtvlstprasrvttlkrtavvlalvaygahklyplvrqclapargpqapave--------  52
Aardvark                mpvlstprpsrvttlkrtavvlaltaygahklyplvrqclapargtlgpvgqplqea---  57
Alpaca                  mtvlstyrpsrvttlkrtavvlaltaygahkvyplvrqclalarspqgsagesaqevsga  60
Armadillo               mpvlstprssrvttlrrtavvlaltaygahkiyplvrqclvparspqgpsrepaqdaaga  60
Baboon                  mpvlsrprpwrgstlkrtavllalaaygvhkvyplvrqclapargsqapareptqeasga  60
Bat                     mpvlstprssrvttlkrtavvlaltaygahkiyplvrqclapargpqvpagqstqeasgt  60
Cat                     mpvlstprpsrvttlkrtalvlaltaygahkiyplvrqylaasrgpqvpagestqdvsga  60
Chinchilla              mpvlstprpsrvttlrrtamvlaltaygahklyplvrqclapargpqgpageppqealgs  60
Chinese-hamster         mpvlstprssrvttlkrtavvlaltaygvhkiyplvrqcltpargpqvpageptqetsgv  60
Cow                     mtvlstprasrvttlkrtavvlalvaygahklyplvrqclapargpqapaveptpdvsga  60
Dog                     mpvlstprssrvttlkrtalvlaltaygahkiyplvrhylaatrgpqvpagestqevsga  60
Dolphin                 ------------------------------------------------------------  0
Ferret                  mpvlstprssrvstlkrtalvlaltaygahkvypllrqylaatrgpqvpagestqevsga  60
Florida-manatee         msvlstprpsrvttlkrtavvlaltaygahkiyplvrqclapargpprpageptqeasga  60
Flying-Fox              mpvlstprlsrvttlkrtavvlaltaygahkiypllrqclaparapqlpagestqevsga  60
Golden-hamster          mpvlstprssrvttlkrtavvlaltaygvhkiyplvrqcltpsrgpqvpaed--qetsgv  58
Ground-Squirrel         mpvlstprpsrvtmlrrtavvlaltaygvhkiyplvrqclassrgpqvptgdptqealea  60
Guinea-pig              mpvlstprpsrvttlrrtavvlaltaygahklyplvrqclapargpqgpageptlealgp  60
Hedgehog                mpvlstpkpsrattlkrtavvlalaaygahklyplarqclalarappgpagdpqpeal-t  59
Horse                   mpvlstpqpsrvttlkrtavvlaltaygahkiyplvrqclapargpqvpagestqevsga  60
Lemur                   mpvlstprpsrvttlkrtavalaitaygvhkiyplvrqclapargpqvpveeptqetsga  60
Minke-whale             --------------------vlaltaygahkiyplvrqclapargpqtpagespqeasga  40
Mole-rat                mpvlstprssrvitlkrtavvlaltayglqkiyplvrqcltpargaqmpageptqqasga  60
Naked-mole-rat          mpvlstprpsrvttlrrtavvlaltaygahklyplvrqcmtpargpqgpageslqeapep  60
Opposum                 ------------------------------------------------------------  0
Orca                    mtvlstprpsrvttlkrtavvlaltaygahkiyplvrqclapargpqtpagesaqeasga  60
Prairie-vole            mpvlstprssrvttlkrtavvlaltaygvhkiyplvrqcltpargpqvpageptqeasga  60
Python                  -----------------------------------------------------------a  1
Small-eared-galago      mpvlstprssrvttlkrtavvlaltaygvhkiyplvrqclaparvtqgptreptqevsgs  60
Sperm-whale             mtvlstprpsrvttlkrtavvlaltaygahkiyplvrqclapargpqtpagesaqeasga  60


Human                   aaakagmnrvflqrllwllrllfprvlcretgllalhsaalvsrtflsvyvarldgrlar  120
Chimpanzee              aaakagmnrvflqrllwllrllfprvlcretgllalhsaalvsrtflsvyvarldgrlar  120
Elephant                aaakasvnrvflqrllwllrllfprvlcretgllalhsaalvsrtflsvyvarldgrlar  120
Gorilla                 aaakagmnrvflqrllwllrllfprvlcretgllalhsaalvsrtflsvyvarldgrlar  120
Green-Monkey            --akagmnrvflqrllwllrllfprvlcretgllalhsaalvsrtflsvyvarldgrlar  116
Jerboa                  taakagmnrvflqrllvllrllfpqvlcretgllalhsaalvsrtflsvyvarldgrlar  120
Macaque                 aaakagmnrvflqrlmwllrllfprvlcretgllalhsaalvsrtflsvyvarldgrlar  120
Marmoset                aaaktgmnrvflqrllwllrllfprvlcretgllalhsaalvsrtflsvyvarldgrlar  120
Mouse                   tatkagmnrvflqrllallrllfprvlcretgllalhsaalvsrtflsvyvarldgrlar  120
Orangutan               aaakagmnrvflqrllwllrllfprvlcretgllalhsaalvsrtflsvyvarldgrlar  120
Panda                   taakasvnrvflqrllgllrllfprtlcretgllalhsaslvsrtflsvyvarldgrlar  120
Pig                     taakagvnrvflqrllwllrllfprilcretgllalhsaalvsrtflsvyvarldgrlar  120
Pika                    taakagmnrvflqrllwllrllfprvlcketgllalhstalvsrtflsvyvarldgrlar  120
Prairie-deer-mouse      taaragmnrvflqrllvllrllfprvlcretgllalhsaalvsrtflsvyvarldgrlar  120
```

```
Rabbit                  taakagmnrvflqrllwllrllfprvlcretgllalhsaalvsrtflsvyvarldgrlar  120
Rat                     ttakagmnrvflqrllvllrllfpgvlcretgllalhsaalvsrtflsvyvarldgrlar  120
Rhesus-Monkey           aaakagmnrvflqrlmwllrllfprvlcretgllalhsaalvsrtflsvyvarldgrlar  120
Seal                    taakagvnrvflqrllgllrllfprtlcretgllalhsaslvsrtflsvyvarldgrlar  120
Squirrel-Monkey         aaakagmnrvflqrllwllrllfprvlcretgllalhsaalvsrtflsvyvarldgrlar  120
Tarsier                 saakagmnrvflqrllwllrllfprvlcretgllalhsgalvsrtflsvyvarldgrlar  120
Tasmanian-devil         ------------------------------------------------------------  0
Walrus                  taakagvnrvflqrllgllrllfprtlcretgllalhsaslvsrtflsvyvarldgrlar  120
Water-buffalo           ---kagvnqvflqrllwllrllfprilcretgllalhsaalvsrtflsvyvarldgrlar  57
White-Rhinoceros        -aakagvnrvflqrllwllrllfprilcretgllalhsatlvsrtflsvyvarldgrlar  119
Yak                     ----------flqrllwllrllfprilcretgllalhsaalvsrtflsvyvarldgrlar  102
Aardvark                ---kasvnrvflqrllwllrllfprvlcretgllalhsaalvsrtflsvyvarldgrlar  114
Alpaca                  t--kagvnrvflqrllwllrllfprilcretgllalhsaalvsrtflsvyvarldgrlar  118
Armadillo               aaakasvnrvflrrllwllrllfprvlcretgllalhsaalvsrtflsvyvarldgrlvr  120
Baboon                  aaakagmnrvflqrllwllrllfprvlcretgllalhsaalvsrtflsvyvarldgrlar  120
Bat                     tgakagvnrvflqrllwllrllfprilcretgllalhsaalvsrtflsvyvarldgrlar  120
Cat                     taakasvnrvflrrllgllrllfprtlcretgllalhsaslvsrtflsvyvarldgrlar  120
Chinchilla              taakagmnrvflrrllvllrllfprvlcretgllalhsaalvsrtflsvyvarldgrlar  120
Chinese-hamster         taakagmnrvflqrllvllrllfprilcretgllalhsaalvsrtflsvyvarldgrlar  120
Cow                     pagkagvnqvflqrllwllrllfprilcretgllalhsaalvsrtflsvyvarldgrlar  120
Dog                     taakasvnrvflqrllgllrllfprtlcretgllalhsaslvsrtflsvyvarldgrlar  120
Dolphin                 -------nrvffqrllwllrllfprilcretgllalhsaalvsrtflsvyvarldgrlar  53
Ferret                  taakasvnrvflqrlfgllrllfprtlcretgllalhsaslvsrtflsvyvarldgrlar  120
Florida-manatee         a-akasvnrvflqrllwllrllfprvlcretgllalhsaalvsrtflsvyvarldgrlar  119
Flying-Fox              tatkagvnrvflqrllwllrllfprilcretgllvlhsaalvsrtflsvyvarldgrlar  120
Golden-hamster          taakagmnrvflqrllvllrllfprilcretgllalhsaalvsrtflsvyvarldgrlar  118
Ground-Squirrel         taakagmnrvflqrllvllrllfprvlcretallalhsaalvsrtflsvyvarldgrlar  120
Guinea-pig              taakagmnrvflrrllvllrllfprvlcretgllalhsaalvsrtflsvyvarldgrlar  120
Hedgehog                taakasvnraflqrllwllrlifprllcretgllalhsatlvsrtflsvyvaqldgrlar  119
Horse                   taakagvnrvflqrllwllrllfpkilcretgllalhsatlvsrtflsvyvarldgrlar  120
Lemur                   tgakagmnqvflqrllwllrllfprvlcretgllalhsaalvsrtflsvyvarldgrlar  120
Minke-whale             taakagvnrvffqrllwllrllfprilcretgllalhsaalvsrtflsvyvarldgrlar  100
Mole-rat                taaktgmnrvflqrllvllrllfprvlcretgllalhsaalvsrtflsvyvarldgrlar  120
Naked-mole-rat          tatkagmnrvflrrflvllrllfpqvlcretgllalhsaalvsrtflsvyvarldgrlar  120
Opposum                 -----------------llrllfpkvlcretallglhtaalvsrtflsvyvarldgrlar  43
Orca                    taakagvnrvffqrllwllrllfprilcretgllalhsaalvsrtflsvyvarldgrlar  120
Prairie-vole            taakagmnrvflqrllvllrllfprvlcretgllalhstalvsrtflsvyvarldgrlar  120
Python                  tasppgvnkvfflrllrllrllfpgplcrqtgllalhsaalasrtflsvyvaqldgrlar  61
Small-eared-galago      tvakagmnrvflqrllwllrllfprvvcretgllalhsaalvsrtflsvyvarldgrlar  120
Sperm-whale             taakagvnrvffqrllwllrllfprilcretgllalhsaalvsrtflsvyvarldgrlar  120


Human                   civrkdprafgwqllqwllialpatfvnsairylegqlalsfrsrlvahayrlyfsqqty  180
Chimpanzee              civrkdprafgwqllqwllialpatfvnsairylegqlalsfrsrlvahayrlyfsqqty  180
Elephant                civrkdprafgwqllqwllialpatfinsairylegqlalsfrsrlvahayslyfsqqty  180
Gorilla                 civrkdprafgwqllqwllialpatfvnsairylegqlalsfrsrlvahayrlyfsqqty  180
Green-Monkey            civrkdprafgwqllqwllialpatfvnsairylegqlalsfrsrlvahayrlyfsqqty  176
Jerboa                  civrkdprafswqllqwllialpatfvnsairylegqlalsfrsrlvahayslyfsqqty  180
Macaque                 civrkdprafgwqllqwllialpatfvnsairylegqlalsfrsrlvahayrlyfsqqty  180
Marmoset                civrkdprafgwqllqwllialpatfvnsairylegqlalsfrsrlvahayslyfsqqty  180
Mouse                   civrkdprafswqllqwllialpatfinsairylegqlalsfrsrlvahayglyfsqqty  180
Orangutan               civrkdprafgwqllqwllialpatfvnsairylegqlalsfrsrlvahayrlyfsqqty  180
Panda                   civrkdpqafgwqllqwllialpatfinsairylegqlalsfrsrlvahayslyfsqqty  180
Pig                     civrkdprafgwqllqwlfialpatfvnsairylegqlalafrsrlvahayslyfsqqty  180
Pika                    civrkdprafswqllqwllialpatfinsairylegqlalafrsrlvahayslyfsqqty  180
Prairie-deer-mouse      civrkdprafswqllqwllialpatfinsairylegqlalsfrsrlvahayslyfsqqty  180
Rabbit                  civrkaprafswqllqwllialpatfinsairylegqlalsfrsrlvahayslyfsqqty  180
Rat                     civrkdprafswqllqwllialpatfinsairylegqlalsfrsrlvahayglyfsqqty  180
Rhesus-Monkey           civrkdprafgwqllqwllialpatfvnsairylegqlalsfrsrlvahayrlyfsqqty  180
Seal                    civrkdpqafgwqllqwllialpatfinsairylegqlalsfrsrlvahayrlyfsqqty  180
Squirrel-Monkey         civrkdprafgwqllqwllialpatfvnsairylegqlalsfrsrlvahayslyfsqqty  180
Tarsier                 civrkdprafgwqllqwlfialpatfinsairylegqlalsfrsrlvahayslyfaqqty  180
Tasmanian-devil         -ivrkqprafswqllqwllialpatfvnsairylegrlalafrsrlvghayrlyfnqqty  59
Walrus                  civrkdpqafgwqllqwllialpatfinsairylegqlalsfrsrlvahayrlyfsqqty  180
Water-buffalo           civrkdpqafswqlvqwllialpatfinsairylegqlalafrsrlvahayslyfsqqty  117
White-Rhinoceros        civrkdprafgwqllqwllialpatfinsairylegqlalsfrsrlvahayslyfsqqty  179
Yak                     civrkdpqafswqlvqwllialpatfinsairylegqlalafrsrlvahayslyfsqqty  162
Aardvark                civrkdprafgwqllqwllialpatfvnsairylegqlalsfrsrlvahayglyfsqqty  174
Alpaca                  civrkdprafgwqllqwllialpatfvnsairylegqlalsfrsrlvahayslyfsqqty  178
Armadillo               civrkdprafswqllqwlfialpatfvnsairylegqlalafrsrlvahayrlyfsqqty  180
Baboon                  civrkdprafgwqllqwllialpatfvnsairylegqlalsfrsrlvahayrlyfsqqty  180
Bat                     civrkdpqafgwqllqwilialpatfinsairflegqlalsfrsrlvahayslyfsqqty  180
Cat                     civrkdprafswqllqwllialpatfinsairylegqlalsfrsrlvahayslyfsqqty  180
Chinchilla              civrkdprafgwqllqwllialpatfvnsairylegqlalsfrsrlvahayslyfsqqty  180
Chinese-hamster         civrkdprafswqllqwllialpatfinsairylegqlalsfrsrlvahayslyfsqqty  180
Cow                     civrkdpqafswqlvqwllialpatfinsairylegqlalafrsrlvahayslyfsqqty  180
Dog                     civrkdprafgwqllqwllialpatfinsairylegqlalsfrsrlvahayslyfsqqty  180
```

```
Dolphin             civrkdprafgwqllqwllialpatfvnsairylegrlalafrsrlvahayslyfsqqty  113
Ferret              givrkdprafgwqllqwllialpatfinsairflegqlalsfrsrlvahayslyfsqqty  180
Florida-manatee     civrkdprafgwqllqwllialpatfinsairylegqlalsfrsrlvahayslyfsqqty  179
Flying-Fox          civrkdprafgwqllqwllialpatfinsairflegqlalsfrsrlvahayslyfsqqty  180
Golden-hamster      civrkdprafswqllqwllialpatfinsairylegqlalsfrsrlvahayslyfshqty  178
Ground-Squirrel     civrkdprafswqllqwllialpatfinsairylegqlalsfrsrlvahayslyfsqqty  180
Guinea-pig          civrkdprafgwqllqwllialpaxfvnsairylegqlalsfrsrlvthayslyfsqqty  180
Hedgehog            civrkdphafgwqllqwllialpatfinsairylegqlalafrsrlvahayslyfsqqty  179
Horse               civrkdpqafgwqllqwllialpatfinsairylegqlalsfrsrlvahayslyfsqqty  180
Lemur               civrkdprafswqllqwllialpatfinsairylegqlalsfrsrlvahayslyfsqqty  180
Minke-whale         civrkdprafgwqllqwllialpatfvnsairylegrlalafrsrlvahayslyfsqqty  160
Mole-rat            civrkdprafswrllqwllialpatfinsairylegqlalafrsrlvahayslyfsqqty  180
Naked-mole-rat      civrkdprafgwqlvqwllialpatfvnsairylegqlalsfrsrlvthayslyfsqqty  180
Opposum             civrkqprafswqllqwllialpatfvnsairylegrlalafrsrlvghayrlyfsrqty  103
Orca                civrkdprafgwqllqwllialpatfvnsairylegrlalafrsrlvahayslyfsqqty  180
Prairie-vole        civrkdpqafswqllqwllialpatfinsairylegqlalsfrsrlvahayrlyfsqqty  180
Python              civrknprdftwqllqwllialpatfvnsairylegqlslafrgrlvdhayqlyfegqty  121
Small-eared-galago  civrkdpqafgwqllqwllialpatfinsairylegqlalsfrsrlvahayslyfsqqty  180
Sperm-whale         civrkdprafgwqllqwllialpatfvnsairylegrlalafrsrlvahayslyfsqqty  180


Human               yrvsnmdgrlrnpdqsltedvvafaasvahlysnltkplldvavtsytllraarsrgagt  240
Chimpanzee          yrvsnmdgrlrnpdqsltedvvafaasvahlysnltkplldvavtsytllraarsrgagt  240
Elephant            yrvsnmdgrlrnpdqsltedlvafaasvahlysnltkplldvavtsytllraarsrgagt  240
Gorilla             yrvsnmdgrlrnpdqsltedvvafaasvahlysnltkplldvavtsytllraarsrgagt  240
Green-Monkey        yrvsnmdgrlrnpdqsltedvvafaasvahlysnltkplldvavtsytllqvarscgagt  236
Jerboa              yrvsnmdgrlrnpdqsltedvvafaasvahlysnltkplldvavtsytllraarsrgagt  240
Macaque             yrvsnmdgrlrnpdqsltedvvafaasvahlysnltkplldvavtsytllqaarsrgagt  240
Marmoset            yrvsnmdgrlrnpdqsltedvvafaasvahlysnltkplldvavtsytllraarsrgagt  240
Mouse               yrvsnmdgrlrnpdqsltedvvafaasvahlysnltkplldvavtsytllraarsrgagt  240
Orangutan           yrvsnmdgrlrnpdqsltedvvafaasvahlysnltkplldvavtsytllraarsrgagt  240
Panda               yrvsnmdgrlrnpdqsltedvvafaasvahlysnltkplldvavtaytllraarsrgagt  240
Pig                 yrvsnmdgrlrnpdqsltedvvafaasvahlysnltkplldvavtaytllraarsrgagt  240
Pika                yrvsnmdgrlrnpdqsltedvvafaasvahlysnltkplldvavtsytllraarsrgagt  240
Prairie-deer-mouse  yrvsnmdgrlrnpdqsltedvvafaasvahlysnltkplldvavtsytllraarsrgagt  240
Rabbit              yrvsnmdgrlrnpdqsltedvvafaasvahlysnltkplldvavtsytllraarsrgagt  240
Rat                 yrvsnmdgrlrnpdqsltedmvafaasvahlysnltkplldvavtsytllraarsrgagt  240
Rhesus-Monkey       yrvsnmdgrlrnpdqsltedvvafaasvahlysnltkplldvavtsytllqaarsrgagt  240
Seal                yrvsnmdgrlrnpdqsltedvvafaasvahlysnltkplldvavtaytllraarsrgagt  240
Squirrel-Monkey     yrvsnmdgrlrnpdqsltedvvafaasvahlysnltkplldvavtsytllraarsrgagt  240
Tarsier             yrvsnmdgrlrnpdqsltedvvafaasvahlysnltkplldvavtsytllraarsrgagt  240
Tasmanian-devil     yrvsnmdgrlrnpdqsltedvvafaasvahlysnltkplldvavtsytllrtarsrgast  119
Walrus              yrvsnmdgrlrnpdqsltedvvafaasvahlysnltkplldvavtaytllraarsrgagt  240
Water-buffalo       yrvsnmdgrlrnpdqsltedvvafaasvahlysnltkplldvavttytlvraarsrgagt  177
White-Rhinoceros    yrvsnmdgrlrnpdqsltedvvafaasvahlysnltkplldvavtaytllraarsrgagt  239
Yak                 yrvsnmdgrlrnpdqsltedvvafaasvahlysnltkplldvavttytlvraarsrgagt  222
Aardvark            yrvsnmdgrlrnpdqsltedvvafaasvahlysnltkplldvavtsytllraarsrgagt  234
Alpaca              yrvsnmdgrlrnpdqsltedvvafaasvahlysnltkplldvavtaytllrearsrgagt  238
Armadillo           yrvsnmdgrlrnpdqsltedvvafaasmahlysnltkplldvavtsytliqaarsrgagt  240
Baboon              yrvsnmdgrlrnpdqsltedvvafaasvahlysnltkplldvavtsytllqaarsrgagt  240
Bat                 yrvsnmdgrlrnpdqsltedvvtfaasvahlysnltkplldvavtaytllrtarsrgagt  240
Cat                 yrvsnmdgrlrnpdqsltedvvafaasvahlysnltkplldvavtaytlvraarsrgagt  240
Chinchilla          yrvsnmdgrlrnpdqsltedlvafaasvahlysnltkplldvavtsytliraarsrgagt  240
Chinese-hamster     yrvsnmdgrlrnpdqsltedvvafaasvahlysnltkplldvavtsytllraarsrgagt  240
Cow                 yrvsnmdgrlrnpdqsltedvvafaasvahlysnltkplldvavttytlvraarsrgagt  240
Dog                 yrvsnmdgrlrnpdqsltedvvafaasvahlysnltkplldvavtaytllraarsrgagt  240
Dolphin             yrvsnmdgrlrnpdqsltedvvafaasvahlysnltkplldvavttytlvrvarsrgagt  173
Ferret              yrvsnmdgrlrnpdqsltedvvafaasvahlysnltkplldvavtaytllraarsrgagt  240
Florida-manatee     yrvsnmdgrlrnpdqsltedvvafaasvahlysnltkplldvavtaytllraarsrgagt  239
Flying-Fox          yrvsnmdgrlrnpdqsltedvvafaasvahlysnltkplldvavtaytliqtarsrgagt  240
Golden-hamster      yrvsnmdgrlrnpdqsltedvvafaasvahlysnltkplldvavtsytllraarsrgagt  238
Ground-Squirrel     yrvsnmdgrlrnpdqsltedvvafaasvahlysnltkplldvavtsytllraarsrgagt  240
Guinea-pig          yrvsnmdgrlrnpdqsltedlvafaasvahlysnltkplldvavtsytlvraarsrgagt  240
Hedgehog            yrvsnmdgrlrnpdqsltedvvafaasvahlysnltkplldvavtaytllraarsrgagt  239
Horse               yrvsnmdgrlrnpdqsltedvvafaasvahlysnltkplldvavtaytllraarsrgagt  240
Lemur               yrvsnmdgrlrnpdqsltedvvafaasvahlysnltkplldvavtsytllraarsrgagt  240
Minke-whale         yrvsnmdgrlrnpdqsltedvvafaasvahlysnltkplldvavttytlvrvarsrgagt  220
Mole-rat            yrvsnmdgrlrnpdqsltedvvafaasvahlysnltkplldvavtsytllraarsrgagt  240
Naked-mole-rat      yrvsnmdgrlrnpdqsltedlvaftasvahlysnltkplldvavtsytliraarsrgagt  240
Opposum             yrvsnmdgrlrnpdqsltedlvafaasvahlysnltkplldvavtsytllrtarsrgast  163
Orca                yrvsnmdgrlrnpdqsltedvvafaasvahlysnltkplldvavttytlvrvarsrgagt  240
Prairie-vole        yrvsnmdgrlrnpdqsltedvvafaasiahlysnltkplldvavtsytllraarsrgagt  240
Python              yrvsnmdgrlrnpdqsltedlvafansmahlysnltkpvldlivtsytllssarskgadt  181
Small-eared-galago  yrvsnmdgrlrnpdhsltedvvafaasvahlysnltkplldvavtsytllraarsrgagt  240
Sperm-whale         yrvsnmdgrlrnpdqsltedvvafaasvahlysnltkplldvavttytlvrvarsrgagt  240
```

```
Human                    awpsaiaglvvfltanvlrafspkfgelvaeearrkgelrymhsrvvanseeiafygghe  300
Chimpanzee               awpsaiaglvvfltanvlrafspkfgelvaeearrkgelrymhsrvvanseeiafygghe  300
Elephant                 awpsaiaglvvfltanvlrafspkfgelvaeearrkgelrymhsrvvanseeiafygghe  300
Gorilla                  awpsaiaglvvfltanvlrafspkfgelvaeearrkgelrymhsrvvanseeiafygghe  300
Green-Monkey             awpsaiaglvvfltanvlrafspkfgelvaeearrkgelrymhsrvvanseeiafygghe  296
Jerboa                   awpsaiaglvvfltanvlrafspkfgelvaeearrkgelrymhsrvvanseeiafygghe  300
Macaque                  awpsaiaglvvfltanvlrafspkfgelvaeearrkgelrymhsrvvanseeiafygghe  300
Marmoset                 awpsaiaglvvfltanvlrafspkfgelvaeearrkgelrymhsrvvanseeiafygghe  300
Mouse                    awpsaiaglvvfltanvlrafspkfgelvaeearrkgelrymhsrvvanseeiafygghe  300
Orangutan                awpsaiaglvvfltanvlrafspkfgelvaeearrkgelrymhsrvvanseeiafygghe  300
Panda                    awpsaiaglvvfltanvlrafspkfgelvaeearrkgelrymhsrvvanseeiafygghe  300
Pig                      awpsaiaglvvfltanvlrafspkfgelvaeearrkgelrymhsrvvanseeiafygghe  300
Pika                     awpsaiaglvvfltanvlrafspkfgelvaeearrkgelrymhsrvvanseeiafygghe  300
Prairie-deer-mouse       awpsaiaglvvfltanvlrafspkfgelvaeearrkgelrymhsrvvanseeiafygghe  300
Rabbit                   awpsaiaglvvfltanvlrafspkfgelvaeearrkgelrymhsrvvanseeiafygghe  300
Rat                      awpsaiaglvvfltanvlrafspkfgelvaeearrkgelrymhsrvvanseeiafygghe  300
Rhesus-Monkey            awpsaiaglvvfltanvlrafspkfgelvaeearrkgelrymhsrvvanseeiafygghe  300
Seal                     awpsaiaglvvfltanvlrafspkfgelvaeearrkgelrymhsrvvanseeiafygghe  300
Squirrel-Monkey          awpsaiaglvvfltanvlrafspkfgelvaeearrkgelrymhsrvvanseeiafygghe  300
Tarsier                  awpsaiaglvvfltanvlrafspkfgelvaeearrkgelrymhsrvvanseeiafygghe  300
Tasmanian-devil          awpsaiaglvvfltakvlracspkfgelvaeearrkgelrymhsrvvanseeiafygghe  179
Walrus                   awpsaiaglvvfltanvlrafspkfgelvaeearrkgelrymhsrvvanseeiafygghe  300
Water-buffalo            awpsaiaglvvfltanvlrafspkfgelvaeearrkgelrymhsrvvanseeiafygghe  237
White-Rhinoceros         awpsaiaglvvfltanvlrafspkfgelvaeearrkgelrymhsrvvanseeiafygghe  299
Yak                      awpsaiaglvvfltanvlrafspkfgelvaeearrkgelrymhsrvvanseeiafygghe  282
Aardvark                 awpsaiaglvvfltanilrafspkfgelvaeearrkgelrymhsrvvanseeiafygghe  294
Alpaca                   awpsaiaglvvfltanvlrafspkfgelvaeearrkgelrymhsrvvanseeiafygghe  298
Armadillo                awpsaiaglavfltanvlrafspkfgelvaeearrkgelrymhsrvvanseeiafygghe  300
Baboon                   awpsaiaglvvfltanvlrafspkfgelvaeearrkgelrymhsrvvanseeiafygghe  300
Bat                      awpsaiaglvvfltanvlrafspkfgelvaeearrkgelrymhsrvvanseeiafygghe  300
Cat                      awpsaiaglvlfltanvlrafspkfgelvaeearrkgelrymhsrvvanseeiafygghe  300
Chinchilla               awpsaiaglvvfltanvlrafspkfgelvaeearrkgelrymhsrvvanseeiafygghe  300
Chinese-hamster          awpsaiaglvvfltanvlrafspkfgelvaeearrkgelrymhsrvvanseeiafygghe  300
Cow                      awpsaiaglvvfltanvlrafspkfgelvaeearrkgelrymhsrvvanseeiafygghe  300
Dog                      awpsaiaglvvfltanvlrafspkfgelvaeearrkgelrymhsrvvanseeiafygghe  300
Dolphin                  awpsaiaglvvfltanvlqafspkfgelvaeearrkgelrymhsrvvanseeiafygghe  233
Ferret                   awpsaiaglvvfltanvlrafspkfgelvaeearrkgelrymhsrvvanseeiafygghe  300
Florida-manatee          awplaiaglvvfltanvlrtfspkfgelvaeearrkgelrymhsrvvanseeiafygghe  299
Flying-Fox               awpsaiaglvvfltanvlrafspkfgelvaeearrkgelrymhsrvvanseeiafygghe  300
Golden-hamster           awpsaiaglvvfltanvlrafspkfgelvaeearrkgelrymhsrvvanseeiafygghe  298
Ground-Squirrel          awpsaiaglvvfltanvlrafspkfgelvaeearrkgelrymhsrvvanseeiafygghe  300
Guinea-pig               awpsaiaglvvfltanvlrasspkfgelvaeearrkgelrymhsrvvanseeiafygghe  300
Hedgehog                 awplaiaglvvfltanvlrafspkfgelvaeearrkgelrymhsrvvanseeiafygghe  299
Horse                    awpsaiaglvvfltanvlrafspkfgelvaeearrkgelrymhsrvvanseeiafygghe  300
Lemur                    awpsaiaglvvfltanvlrafspkfgelvaeearrkgelrymhsrvvanseeiafygghe  300
Minke-whale              awpsaiaglvvfltanvlrafspkfgelvaeearrkgelrymhsrvvanseeiafygghe  280
Mole-rat                 awpsaiaglvvfltanvlrasspkfgelvaeearrkgelrymhsrvvanseeiafygghe  300
Naked-mole-rat           awpsaiaglvvfltanvlrafspkfgelvaeearrkgelrymhsrvvanseeiafygghk  300
Opposum                  twpsaiaglvvfltakvlracspkfgelvaeearrkgelrymhsrvvanseeiafygghe  223
Orca                     awpsaiaglvvfltanvlqafspkfgelvaeearrkgelrymhsrvvanseeiafygghe  300
Prairie-vole             awpsaiaglvvfltanvlrafspkfgelvaeearrkgelrymhsrvvanseeiafygghe  300
Python                   awpsviaglvvcitakvlracspkfgqlvaeearrkgelrymhsrvvanseeiafygghk  241
Small-eared-galago       awpsaiaglvvfltanvlracspkfgelvaeearrkgelrymhsrvvanseeiafygghk  300
Sperm-whale              awpsaiaglvvfltanvlrafspkfgelvaeearrkgelrymhsrvvanseeiafygghe  300


Human                    velallqrsyqdlasqinlillerlwyvmleqflmkyvwsasgllmvavpiitatgyses  360
Chimpanzee               velallqrsyqdlasqinlillerlwyvmleqflmkyvwsasgllmvavpiitatgyses  360
Elephant                 velallrhsyqdlasqinlillerlwyvmleqflmkyvwsasgllmvavpiitatgyses  360
Gorilla                  velallqrsyqdlasqinlillerlwyvmleqflmkyvwsasgllmvavpiitatgyses  360
Green-Monkey             velallqhsyqdlasqinlillerlwyvmleqflmkyvwsasgllmvavpiitatgyses  356
Jerboa                   velallqhsyrdlasqinlillerlwyvmleqflmkyvwsasgllmvavpiitatgyses  360
Macaque                  velallqhsyqdlasqinlillerlwyvmleqflmkyvwsasgllmvavpiitatgyset  360
Marmoset                 velallqhsyqdlasqinlillerlwyvmleqflmkyvwsasgllmvavpiitatgyses  360
Mouse                    velallqhsyqdlasqinlillerlwyvmleqflmkyvwsasgllmvavpiitatgyaes  360
Orangutan                velallqrsyqdlasqinlillerlwyvmleqflmkyvwsasgllmvavpiitatgyses  360
Panda                    velallqhsyqdlasqinlillerlwyimleqflmkyvwsasgllmvavpiitatgyses  360
Pig                      velallqhsyqdlasqinlillerlwyvmleqflmkyvwsasgllmvavpiitatgyses  360
Pika                     velallrrsyqdlasqihlillerlwyvmleqflmkyvwsasgllmvavpiitatgyses  360
Prairie-deer-mouse       velallqhsyqdlasqinlillerlwyvmleqflmkyvwsasgllmvavpiitatgyaes  360
Rabbit                   velallqrsyqdlasqihlillerlwyvmleqflmkyvwsasgllmvavpiitatgyses  360
Rat                      velallqhsyqdlasqinlillerlwyvmleqflmkyvwsasgllmvavpiitatgyaes  360
Rhesus-Monkey            velallqhsyqdlasqinlillerlwyvmleqflmkyvwsasgllmvavpiitatgyset  360
Seal                     velallqhsyqdlasqinlillerlwyimleqflmkyvwsasgllmvavpiitatgyses  360
Squirrel-Monkey          velallqhsyqrlasqinlillerlwyvmleqflmkyvwsasgllmvavpiitatgyses  360
Tarsier                  velallqhsyqdlasqinlillerlwyvmleqflmkyvwsasgllmvavpiitatgyses  360
```

```
Tasmanian-devil        velallqhsyqdlasqinlillerlwyvmleqflmkyvwsasglvmvavpiitavgyset  239
Walrus                 velallqhsyqdlasqinlillerlwyimleqflmkyvwsasgllmvavpiitatgyses  360
Water-buffalo          velallqhsyqdlasqinlillerlwyvmleqflmkyvwsasgllmvavpiitatgyses  297
White-Rhinoceros       velallqhsyqdlasqinlillerlwyvmleqflmkyvwsasgllmvavpiitatgyses  359
Yak                    velallqhsyqdlasqinlillerlwyvmleqflmkyvwsasgllmvavpiitatgyses  342
Aardvark               velallrlsyqdlasqinlillerlwyvmleqflmkyvwsasgllmvavpiitatgysea  354
Alpaca                 velallqhsyqdlasqinlillerlwyvmleqflmkyvwsasglvmvavpiitatgyses  358
Armadillo              velallqqsyrdlasqinlillerlwyvmleqflmkyvwsasglvmvavpiitatgfses  360
Baboon                 velallqhsyqdlasqinlillerlwyvmleqflmkyvwsasgllmvavpiitatgyses  360
Bat                    velallqhsykalasqinlillerlwyvmleqflmkyvwsasgllmvavpiitatgfses  360
Cat                    velallrhsyqdlasqinlillerlwyimleqflmkyvwsasgllmvavpiitatgyses  360
Chinchilla             velallrdcyralaaqinvillerlwyvmleqflmkyvwsasgllmvavpiitatgyses  360
Chinese-hamster        velallrqsyqdlasqinlillerlwyvmleqflmkyvwsasgllmvavpiitatgyaes  360
Cow                    velallqhsyqdlasqinlillerlwyvmleqflmkyvwsasgllmvavpiitatgyses  360
Dog                    velallqhsyqdlasqinlillerlwyimleqflmkyvwsasgllmvavpiitatgyses  360
Dolphin                velallqhsyqdlasqinlillerlwyvmleqflmkyvwsasgllmvavpiitatghaes  293
Ferret                 velallqqsyqglasqihlillerlwyitleqflmkyvwsasgllmvavpiitatgyaes  360
Florida-manatee        velallqhsyeelasqinlillerlwyvmleqflmkyvwsasgllmvavpiitatgyses  359
Flying-Fox             velallqhsykalasqinlillerlwyvmleqflmkyvwsasgllmvavpiitatgyses  360
Golden-hamster         velallqqsyqdlasqinlillerlwyvmleqflmkyvwsasgllmvavpiitatgyaes  358
Ground-Squirrel        velaqlqhsyqnlasqinlillerlwyvmleqflmkyvwsasgllmvavpiitatgyses  360
Guinea-pig             velallrdcyralaaqinvillkrlwyvmleqflmkyvwsasgllmvavpiitatgyses  360
Hedgehog               veltllqlsykdlashinlillerlwyvmleqflmkyvwsasgllmvavpiitatgyses  359
Horse                  velallqhsyqdlasqinlillerlwyvmleqflmkyvwsasgllmvavpiitatgysea  360
Lemur                  velallqhsyqdlasqinlillerlwyvmleqflmkyvwsasgllmvavpiitatgyses  360
Minke-whale            velallqhsyqdlasqinlillerlwyvmleqflmkyvwsasgllmvavpiitatgyses  340
Mole-rat               velallqhsyrdlasqinlillerlwyimleqflmkyvwsasgllmvavpiitatgyses  360
Naked-mole-rat         velallqdcyralaaqinvillerlwyvmleqflmkyvwsasgllmvavpiitatgyses  360
Opposum                velallrhsyqdlasqinlillerlwyvmleqflmkyvwsasglvmvavpiitatgyses  283
Orca                   velallqhsyqdlasqinlillerlwyvmleqflmkyvwsasgllmvavpiitatghaes  360
Prairie-vole           velallqhsyqdlasqinlillerlwyvmleqflmkyvwsasgllmvavpiitatgyaes  360
Python                 velsllrhcyhelasqinvlllerlwyvmleqflmkyvwsaaglvmvavpiitatgyset  301
Small-eared-galago     velallqhsyqdlasqinlillerlwyvmleqflmkyvwsasgllmvavpiitatgyses  360
Sperm-whale            velallqhsyqdlasqinlillerlwyvmleqflmkyvwsasgllmvavpiitatgyses  360


Human                  daeavkkaalekkeeelvserteaftiarnlltaaadaierimssykevtelagytarvh  420
Chimpanzee             daeavkkaalekkeeelvserteaftiarnlltaaadaierimssykevtelagytarvh  420
Elephant               dseavrkaalekreeelvserteaftiarnlltaaadaierimssykevtelagytarvh  420
Gorilla                daeavkkaalekkeeelvserteaftiarnlltaaadaierimssykevtelagytarvh  420
Green-Monkey           aaeavkkaalekkeeelvserteaftiarnlltaaadaierimssykevtelagytaqvh  416
Jerboa                 dseamkkaalekkeeelvserteaftiarnlltaaadaterimssykevtelagytarvh  420
Macaque                daeavkkaalekkeeelvserteaftiarnlltaaadaierimssykevtelagytarvh  420
Marmoset               daeavrksalekkeeelvserteaftiarnlltaaadaterimssykevtelagytarvh  420
Mouse                  dseamkkaalemkeeelvserteaftiarnlltaaadaterimssykevtelagytarvy  420
Orangutan              daeavkkaalekkeeelvserteaftiarnlltaaadaierimssykevtelagytarvh  420
Panda                  dseavkkaalemkeeelvserteaftiarnlltaaadaierimssykevtelagytarvh  420
Pig                    dsevakkaalemreeelvserteaftiarnlltaaadaierimssykevtelagytarvh  420
Pika                   dsedvkkaamekkeeelvserteaftiarnlltaaadaierimssykevtelagytarvh  420
Prairie-deer-mouse     dseamkkaalemkeeelvserteaftiarnlltaaadaterimssykevtelagytarvy  420
Rabbit                 dseavkkaalakkeeelvserteaftiarnlltaaadaierimssykevtelagytarvh  420
Rat                    dseamkkaalemkeeelvserteaftiarnlltaaadaterimssykevtelagytarvy  420
Rhesus-Monkey          daeavkkaalekkeeelvserteaftiarnlltaaadaierimssykevtelagytarvh  420
Seal                   dleavkkaalemkeeelvserteaftiarnlltaaadaierimssykevtelagytarvy  420
Squirrel-Monkey        daeavrksalekkeeelvserteaftiarnlltaaadaterimssykevtelagytarvh  420
Tarsier                dseavkkaalekeeeelvserteaftiarnlltaaadaierimssykevtelagytarvh  420
Tasmanian-devil        dseavkkaalemkeedlvserteaftiarnlltaaadaiervmssykevtelagytarvy  299
Walrus                 dseavkkaalemkeeelvserteaftiarnlltaaadaierimssykevtelagytarvh  420
Water-buffalo          dsetvkkaaqamkegelvserteaftiarnlltaaadaiervmssykevtelagytarvy  357
White-Rhinoceros       aseamkkaaselreeelvserteaftiarnlltaaadaierimssykevtelagytarvh  419
Yak                    dsetvkkaaqamkegelvserteaftiarnlltaaadaiervmssykevtelagytarvy  402
Aardvark               dseavkkaalekreeelvserteaftiarnlltaaadaierimssykevtelagytarvh  414
Alpaca                 dpevvkkaalemkeeelvserteaftiarnlltaaadaierimssykevtelagytarvh  418
Armadillo              dpeavkqaalekeegdlvserteaftiarslltaaadaiervmssykevtelagytarvh  420
Baboon                 daeavkkaalekkeeelvserteaftiarnlltaaadaierimssykevtelagytarvh  420
Bat                    dpeavkkaalamkeeelvserteaftiarnlltaaadaierimssykevtelagytarvh  420
Cat                    dseavkkaalemregelvserteaftiarnlltaaadaterimssykevtelagytarvh  420
Chinchilla             dseatkraalemreeelvserteaftiarnlltaaadaterimssykevtelagytarvh  420
Chinese-hamster        dsetmkkaalemkeeelvserteaftiarnlltaaadaterimssykevtelagytarvy  420
Cow                    dsetvkkaaqamkegelvserteaftiarnlltaaadaiervmssykevtelagytarvy  420
Dog                    dseavkkaalemreeelvserteaftiarnlltaaadaierimssykevtelagytarvh  420
Dolphin                dsevakkaaleareeelvserteaftiarnlltaaadaiervmvsykevtelagytarvh  353
Ferret                 dpeavkkaalqmkeeelvserteaftiarnlltaaadaierimssykevtelagytarvh  420
Florida-manatee        dseavrkaalekkeeelvserteaftiarnlltaaadaierimssykevtelagytarvh  419
Flying-Fox             dteavkkaaldmkeeelvserteaftiarnlltaaadaiervmssykevtelagytarvh  420
Golden-hamster         dseamkkaalemkeeelvserteaftiarnlltaaadaterimssykevtelagytarvy  418
Ground-Squirrel        dsedmkkaalek--eelvserteaftiarnlltaaadaterimssykevtelagytarvh  418
```

```
Guinea-pig           dseaaqraalemqeeelvserteaftiarnlltaaadaterimssykevtelagytarvy  420
Hedgehog             eaeavkkaamenreeelvserteaftiarnlltaaadaierimssykevtelagytarvh  419
Horse                dsealkkaasemreeelvserteaftiarnlltaaadaierimssykevtelagytarvh  420
Lemur                dseamkkavlekrdeelvserteaftiarnlltaaadaierimssykevtelagytarvh  420
Minke-whale          dsevakkaalqtrgeelvserteaftvarnlltaaadaiervmasykevtelagytarvh  400
Mole-rat             dseamkkaalemkeeelvserteaftiarnlltaaadaterimssykevtelagytarvy  420
Naked-mole-rat       dseaekraalemreeelvserteaftiarnlltaaadaterimssykevtelagytarvh  420
Opposum              dseavkkaalemkeedlvserteaftiarnlltaaadaiervmssykeitelagytarvf  343
Orca                 dsevakkaaleareeelvserteaftiarnlltaaadaiervmvsykevtelagytarvh  420
Prairie-vole         dseamkkaalemkeeelvserteaftiarnlltaaadaterimssykevtelagytarvy  420
Python               dseavkqaalemeekewislrteafttarslltaaadatervissykeatelagytarvy  361
Small-eared-galago   dseatkkaalekreeelvserteaftiarnlltaaadaierimssykevtelagytarvh  420
Sperm-whale          dsevakkaaleareeelvserteaftiarnlltaaadaiervmvsykevtelagytarvh  420


Human                emfqvfedvqrchfkrpreledaqagsgtigrsgvrvegplkirgqvvdveqgiicenip  480
Chimpanzee           emfqvfedvhrchfkrpreledaqagsgtigrsgvrvegplkirgqvvdveqgiicenip  480
Elephant             emfqvfkdvqhchfkrpgepedapvgpgavvssgvhvegplkirgqvvdvehgiicenip  480
Gorilla              emfqvfedvqrchfkrpreledaqagsgtigrsgvrvegplkirgqvvdveqgiicenip  480
Green-Monkey         emfqvfedvqrchfkrpkeledaqagsgtigrsgvrvegplkirgqvvdveqgiicenip  476
Jerboa               emfqvfedvqhcrfkrpgefedahtgtgalvrsgvrvegplkiqgqvvdveqgiicenip  480
Macaque              emfqvfedvqrchfkrpreledaqagsgtigrsgvrvegplkirgqvvdveqgiicenip  480
Marmoset             emfqvfadvqrchfkrpgeledtqpgsgtigragvrvegplkiqgqvvdveqgiicenip  480
Mouse                emfqvfedvkhcrfkrtgdleeaqagpgvmvqsgvhvegplkiqgqvvdveqgiicenip  480
Orangutan            emfqvfedvqrchfkrpreledaqagsgtigrsgvrvegplkiqgqvvdveqgiicenip  480
Panda                emfqvfedvqhcrfkrpgeledaqagpgavarsgvrvegllqirgqvvdveqgiicenip  480
Pig                  emfqvfedvqhcrfkrpgepedaqagsgavvrsgvrvegplqirgqvvdvehgivcenip  480
Pika                 emfqvfedvqhcrfkrpgesedaqvgcgtvtrsgvrvdtplkirgrvvdveqgiicenip  480
Prairie-deer-mouse   emfqvfedvqhcrfkrtgdleeaqarpgamvksgvrvegplkiqgqvvdveqgiicenip  480
Rabbit               emfqvfedvqhcrfkrpgepedaqigsgtvarsgvrvdtplkirgqvvdveqgiicenip  480
Rat                  emfqvfedvqhcrfkrtgdleeaqagpgsmvhsgvhiegplkiqgqvvdveqgiicenip  480
Rhesus-Monkey        emfqvfedvqrchfkrpreledaqagsgtigrsgvrvegplkirgqvvdveqgiicenip  480
Seal                 emfqvfedvqhchfkrpgepedpqagpgavarsgvrvegplqirgqvvdveqgiicenip  480
Squirrel-Monkey      emfqvfadvqrchfkrpgeledtqpgsgtigragvrvegplkiqgqvvdveqgiicenip  480
Tarsier              emfqvfedvqhcrfkrpgepedaqagsgamarsgvrlegslkirgqvvdveqgiicenip  480
Tasmanian-devil      emfqvfedvqrgsfkrpgeqedspvrpgalvrhgvrmegplkikgkvvdveqgiicenip  359
Walrus               emfqvfedvqhchfkrpgepedpqagpgavarsgvrvegplqirgqvvdveqgiicenip  480
Water-buffalo        emfqvfedvqqcrfkrpgepedahtgsgaimrsgvrmegplqirgqvvdveqgivcenip  417
White-Rhinoceros     emfqvfddvqhcrfkrpgepedtqvgagavvkpgvrvegplqirgqvvdveqgiicenip  479
Yak                  emfqvfedvqqcrfkrpgeledahtgsgaimrsgirmegplqirgqvvdveqgivcenip  462
Aardvark             emfqvfedvqhcrfkrpgepedapgdsgavvrhgvrvegplkirgqvvdveqgiicenip  474
Alpaca               emfqvfedvqhcrfkrpgepedaqagsgavvrsgtrvegplqirgqvvdveqgivcenip  478
Armadillo            emfqvfkdvqhchfkrpgelekaqggagaivrpgvhmegpprirgqvvdvekgiicenip  480
Baboon               emfqvfedvqrchfkrpreledaqagsgtigrsgvrvegplkirgqvvdveqgiicenip  480
Bat                  emfqvfedvqhcrfkrpgepedaqagagavvrsgvrvegplqirgqvvdveqgivcdnip  480
Cat                  emfqvfedvrhcrfkrpgelestqagagavarsgvrvegplqirgqvvdveqgiicenip  480
Chinchilla           emfqvfedvqhcrfkrpgeledtqagagavarsgvrmegplriqgqvvdveqgiicenip  480
Chinese-hamster      emfqvfedvqhchfkrtgdleeaqakpgamvqsgihvegplkiqgqvvdveqgiicenip  480
Cow                  emfqvfedvqqcrfkrpgepedahtgsgaimrsgirmegplqirgqvvdveqgivcenip  480
Dog                  emfqvfedvqhcrfkrpgeledtqagpgavarsgvhvegplqirgqvvdveqgiicenip  480
Dolphin              emfqvfedvqhcrfkrpgepedtqagsgavvrsgvrvegslqirgqvvdvergivcenip  413
Ferret               emfqvfedvqhcrfkrpgeledtqagpgavakpgvriegplqirgqvvdveqgiicenip  480
Florida-manatee      emfqvfedvqhchfkrpgepedapvgpgavvspgvrvegplkirgqvvdveqgitcenip  479
Flying-Fox           emfqvfeevqhcrfkrpgepedaqagsgavvrsgvrvegplkiqgqvvdveqgiicdnip  480
Golden-hamster       emfqvfedvqhchfkrtgdleetqakpgatvqsgirvegplkiqgqvvdveqgiicenip  478
Ground-Squirrel      emfqvfedvqhcrfkrpgetedsqawcgtmvrsgvrvegplkiqgkvvdveqgiicenip  478
Guinea-pig           emfqvfedvqhcrfkrpgepedtqagtgavvrsgirveaplrirgqvvdveqgiicenip  480
Hedgehog             emfqvfddvqhcrfkrpgeledssvrpgavmrpgvrvegplkilgqvvdveqgiicenip  479
Horse                emfqvfedvqhcrfkrpgepedtqagpgamvksgvrvegplqirgrvvdveqgivcenip  480
Lemur                emfqvfedvqhcrfkrpgepevaqvgsramvrsgvrvegplkirgqvvdveqgiicenip  480
Minke-whale          emfqvfedvrhcrfkrprepedtqagsgavvrsgvrvegslqirgqvvdveqgivcedip  460
Mole-rat             emfqvfedvqhcrfkrpgdpeeahaasgvmvrsgvriegplkiqgqvvdvehgiicenip  480
Naked-mole-rat       emfqvfedvqhchfkrpgepedaqtgagavvrsgvhlegplrirgqvvdveqdiicenip  480
Opposum              emfqvfedvqqgsfkrpgeledsp--ngmlvkhgvrmegplkirgqvvdveqgiicenip  401
Orca                 emfqvfedvqhcrfkrpgepedtqagsgavvrsgvrvegslqirgqvvdveqgivcenip  480
Prairie-vole         emfqvfedvqhcrfkrtgdleeaqarpgamvrsgvrvegplkiqgqvvdveqgiicenip  480
Python               emfqvfedvkccnfrrpgeldegqtragtvmkhgvrvegplqiqghvidvdhgiicenip  421
Small-eared-galago   emfqvfedvqhchfkrpgeledaqpgsgamvrsgvhmegplkiqgqvvdveqgiicenip  480
Sperm-whale          emfqvfedvrhcrfkrpgepedaqagsgavvrsgvrvegslqirgqvvdveqgivcenip  480


Human                ivtpsgevvvaslnirveegmhllitgpngcgksslfrilgglwptyggvlykpppqrmf  540
Chimpanzee           ivtpsgevvvaslnirveegmhllitgpngcgksslfrilgglwptyggvlykpppqrmf  540
Elephant             iitptgevvvaslnirveegmhllitgsngcgksslfwilgglwpayggvlykpppqrmf  540
Gorilla              ivtpsgevvvaslnirveegmhllitgpngcgksslfrilgglwptyggvlykpppqrmf  540
Green-Monkey         iitpsgevvvaslnirveegmhllitgpngcgksslfrilgglwptyggvlykpppqrmf  536
Jerboa               iitptgevvvaslnikveegmhllitgpngcgksslfrilgglwptyggvlykpppqrmf  540
```

```
Macaque                  iitpsgevvvaslnirveegmhllitgpngcgksslfrilgglwptyggvlykpppqrmf  540
Marmoset                 iitpagevvvaslnirveegmhllitgpngcgksslfrilgglwptyggvlykpppqrmf  540
Mouse                    iitptgevvvaslnirveegmhllitgpngcgksslfrilgglwptysgvlykpppqrmf  540
Orangutan                iitpsgevvvaslnirveegmhllitgpngcgksslfrilgglwptyggvlykpppqrmf  540
Panda                    iitptgevvvaslnirveegmhllitgpngcgksslfrilgglwptyggvlykppprrmf  540
Pig                      iitpagevvvaslnirveegmhllitgpngcgksslfrilgglwptyggvlykpppermf  540
Pika                     ivtpagevvvaslsitveegmhllitgpngcgksslfrilgglwptyggvlykpppqrmf  540
Prairie-deer-mouse       iitptgevvvaslnirveegmhllitgpngcgksslfrilgglwptysgvlykpppqrmf  540
Rabbit                   iitptgevvvaslnirveegmhllitgpngcgksslfrilgglwptyggvlykpppqrmf  540
Rat                      iitptgevvvaslnirveegmhllitgpngcgksslfrilgglwptysgvlykpppqrmf  540
Rhesus-Monkey            iitpsgevvvaslnirveegmhllitgpngcgksslfrilgglwptyggvlykpppqrmf  540
Seal                     iitptgevvvaslnirveegmhllitgpngcgksslfrilgglwptyggvlykpppqrmf  540
Squirrel-Monkey          iitptgevvvaslnirveegmhllitgpngcgksslfrilgglwptyggvlckpppqrmf  540
Tarsier                  iitpagevvvaslnirveegmhllitgpngcgksslfrilgglwptyggvlykpppqrmf  540
Tasmanian-devil          iitptgdvvvanlnirveegmhllitgpngcgksslfrilgglwpayagvlykpppqrmf  419
Walrus                   iitptgevvvaslnirveegmhllitgpngcgksslfrilgglwptyggvlykpppqrmf  540
Water-buffalo            iitptgevvvaslnirveegmhllitgpngcgksslfrilgglwptyggvlykpppqrmf  477
White-Rhinoceros         iitptgevvvaslnirveegmhllitgpngcgksslfrilgglwptyggvlykplpqrmf  539
Yak                      iitptgevvvaslnirveegmhllitgpngcgksslfrilgglwptyggvlykpppqrmf  522
Aardvark                 iitptgevvvaslnirveegmhllitgpngcgksslfrilgglwpaysgvlykpppqrmf  534
Alpaca                   iitptgevvvaslnikveegmhllitgpngcgksslfrvlgglwptyggvlykpppqrmf  538
Armadillo                iitpagevvvaslnirveegmhllitgpngcgksslfrilgglwpaysgvlykpppqrmf  540
Baboon                   iitpsgevvvaslnirveegmhllitgpngcgksslfrilgglwptyggvlykpppqrmf  540
Bat                      iitptgevvvaslnirvedgmhllitgpngcgksslfrilgglwptyggvlykpppqrmf  540
Cat                      iitptgevvvsslnirveegmhllitgpngcgksslfrilgglwpayggvlykpppqrmf  540
Chinchilla               iitptgevvvaslnirveegmhllitgpngcgksslfrilgglwptyggvlykpppqrmf  540
Chinese-hamster          iitptgevvvaslnirveegmhllitgpngcgksslfrilgglwptysgvlykpppqrmf  540
Cow                      iitptgevvvaslnirveegmhllitgpngcgksslfrilgglwptyggvlykpppqrmf  540
Dog                      iitptgevvvaslnirveegmhllitgpngcgksslfrilgglwptyggvlykpppqrmf  540
Dolphin                  ivtptgevvvaslnirveegmhllitgpngcgksslfrilgglwptyggvlykpppqrmf  473
Ferret                   iitptgevvvaslnirveegmhllitgpngcgksslfrilgglwptyggvlykpppqrmf  540
Florida-manatee          iitptgevvvaslnirveegmhllitgpngcgksslfrilgglwpayggvlykpppqrmf  539
Flying-Fox               iitptgevvvaslnirveegmhllitgpngcgksslfrilgglwptyggvlykpppqrmf  540
Golden-hamster           iitptgevvvaslnirveegmhllitgpngcgksslfrilgglwptysgvlykpppqrmf  538
Ground-Squirrel          iitptgevvvaslnirveegmhllitgpngcgksslfrilgglwptyggvlykpppqrmf  538
Guinea-pig               iitptgevvvaslnirveegmhllitgpngcgksslfrilgglwptyggvlykpppqrmf  540
Hedgehog                 iitptgevvvaslnirveegmhllitgpngcgksslfrilgglwptyggvlykpppqrmf  539
Horse                    iitpagevvvaslnirveegmhllitgpngcgksslfrilgglwptyggvlykpppqrmf  540
Lemur                    vvtpagevvvaslnirveegmhllitgpngcgksslfrilgglwptyggvlykpppqrmf  540
Minke-whale              ivtpagevvvaslnirveegthllitgpngcgksslfrilgglwptyggvlykpppqrmf  520
Mole-rat                 iitptgevvvaslnirveegmhllitgpngcgksslfrilgglwptyggvlykpppqrmf  540
Naked-mole-rat           iitptgevvvsslnirveegmhllitgpngcgksslfrilgglwptyggvlykpppqrmf  540
Opposum                  iitptgdvvvanlnirveegmhllitgpngcgksslfrilgglwpayagvlykpppqrmf  461
Orca                     ivtptgevvvaslnirveegmhllitgpngcgksslfrilgglwptyggvlykpppqrmf  540
Prairie-vole             iitptgevvvaslnirveegmhllitgpngcgksslfrilgglwptysgvlykpppqrmf  540
Python                   iitptgdvvvtslnirvdegmhllitgpngcgksslfrilgglwptydgtlykppphrmf  481
Small-eared-galago       iitptgevvvaslnmrveegmhllitgpngcgksslfrvlgglwptyggvlykpppqrmf  540
Sperm-whale              vvtptgevvvaslnirveegmhllitgpngcgksslfrilgglwptyggvlykpppqrmf  540


Human                    yipqrpymsvgslrdqviypdsvedmqrkgyseqdleaildvvhlhhilqreggweamcd  600
Chimpanzee               yipqrpymsvgslrdqviypdsvedmqrkgyseqdleaildvvhlhhilqreggweamcd  600
Elephant                 yipcrpymsvgslrdqviypdsvedmqrkghseqhleaildivnlqhilqreggweavcd  600
Gorilla                  yipqrpymsvgslrdqviypdsvedmqrkgyseqdleaildvvhlhhilqreggweamcd  600
Green-Monkey             yipqrpymsvgslrdqviypdsvedmrrkgyseqdleaildivhlhhilqreggweamcd  596
Jerboa                   yipqrpymsvgslrdqviypdsvkdmrrkgyseqqleailnivhlhhilqreggweavcd  600
Macaque                  yipqrpymsvgslrdqviypdsvadmrrkgyseqdleaildivhlhhilqreggweamcd  600
Marmoset                 yipqrpymsvgslrdqviypdsvedmrrkgyseqdleaildivhlhhilqreggweavcd  600
Mouse                    yipqrpymsvgslrdqviypdsaedmrrkgcseqqleailgivhlrhilqreggweavcd  600
Orangutan                yipqrpymsvgslrdqviypdsvedmrrkgyseqdleaildvvhlhhilqreggweamcd  600
Panda                    yipqrpymsvgslrdqviypdsvedmrrkgyseqhleaildivhlnhilqreggweavcd  600
Pig                      yipqrpympvgslrdqviypdsvedmrrkgyseqhleaildivhlnhilqreggweavcd  600
Pika                     yipqrpymsvgslrdqviypdsvadmrrkgyseqhleaildvvhlhhilqreggweavcd  600
Prairie-deer-mouse       yipqrpymsvgslrdqviypdsaedmrrkgcseqqledilgivhlrhilqreggweavcd  600
Rabbit                   yipqrpymsvgslrdqviypdsvedmrrkgyseqhleaildvvhlhhilqreggweavcd  600
Rat                      yipqrpymsvgslrdqviypdsaedmrrkgcseqqleailgivhlrhilqreggweavcd  600
Rhesus-Monkey            yipqrpymsvgslrdqviypdsvadmrrkgyseqdleaildivhlhhilqreggweamcd  600
Seal                     yipqrpymsvgslrdqviypdsvddmrrkgyseqhleaildivhlnhilqreggweavcd  600
Squirrel-Monkey          yipqrpymsvgslrdqviypdsvedmrrkgyseqdleaildivhlhhilqreggweamcd  600
Tarsier                  yipqrpymsvgslrdqviypdsvedmrrkgyseqhletildivhlhhilqreggweavcd  600
Tasmanian-devil          yipqrpymsvgslrdqviypdtiedmkrkgysdhyleaildivnlnhivqreggweascd  479
Walrus                   yipqrpymsvgslrdqviypdsvedmrrkgyseqhleaildivhlnhilqreggweavcd  600
Water-buffalo            yipqrpymsvgslrdqviypdsvedmrrkgyseehlegildivhlnhilqreggweavcd  537
White-Rhinoceros         yipqrpymsvgslrdqviypdsvedmqrkgyseqhleaildivhlnhilqreggweavcd  599
Yak                      yipqrpymsvgslrdqviypdsvedmrrkgyseehlegildivhlnhilqreggweavcd  582
Aardvark                 yvpqrpymslgtlreqviypdsvedmrrkgfsdrdleaildivhlrhilqreggweavcd  594
Alpaca                   yipqrpymsvgsfrdqviypdsvedmrrkgyseqhleaildivhlnhilqreggweavcd  598
```

Armadillo	yipqrpympvgslrdqviypdtaedmrrkgyseqhleailgivhlhhilqreggweavcd	600
Baboon	yipqrpymsvgslrdqviypdsvedmrrkgyseqdleaildivhlhhilqreggweamcd	600
Bat	yipqrpymsvgslrdqviypdsvedmrrkgyseqhleaildivhlnhilqreggweavcd	600
Cat	yipqrpymsvgslrdqviypdsvedmrrkgyseqhleaildivhlnhilqreggweavcd	600
Chinchilla	yipqrpymsvgslrdqviypdsvedmrrkgcseqqleaildivhlnhilqreggwealcd	600
Chinese-hamster	yipqrpymsvgslrdqviypdsaedmrrkgcseqqleailgivhlrhilqreggweavcd	600
Cow	yipqrpymsvgslrdqviypdsvedmrrkgyseehlegildivhlnhilqreggweavcd	600
Dog	yipqrpymsvgslrdqviypdsvedmrrkgyseqhleaildivhlnhilqreggweavcd	600
Dolphin	yvpqrpymsvgslrdqviypdsvedmrrkgfseqhleaildivhlhhilqreggweavcd	533
Ferret	yipqrpympvgslrdqviypdsvedmrrkgyserhleaildivhlnhilqreggweavcd	600
Florida-manatee	yipqrpymsvgslrdqviypdsvedmrrkgyseqhleaildivhlhhilqreggweavcd	599
Flying-Fox	yipqrpymsvgslrdqviypdsvedmrrkgyseqhleaildivhlnhilqreggweavcd	600
Golden-hamster	yipqrpymsvgslrdqviypdsaedmrrkgcseqqleailsivhlrhilqreggweavcd	598
Ground-Squirrel	yipqrpymsvgslrdqviypdsvedmrrkgyseqqleaildivhlhhilqreggweaicd	598
Guinea-pig	yipqrpymsvgslrdqviypdsvedmrrkgcseqqleaildivhlnhilqreggwealcd	600
Hedgehog	yipqrpymsvgslrdqiiypdsvadmlrkgyseqhleaildivhlhhilqreggweavcd	599
Horse	yipqrpymsvgslrdqviypdsvedmrrkgyseqhleaildivhlnhilqreggweatcd	600
Lemur	yipqrpympvgslrdqviypdsvadmrrkgysehhleaildivhlhhilqreggweavcd	600
Minke-whale	yvsrrpymsvgslrdqviypdsvedmrrkgfseqhleaildivhlnhilqreggweavcd	580
Mole-rat	yipqrpymsvgslrdqviypdsvedmrrkgcseqqleaildivhlhhilqreggweavcd	600
Naked-mole-rat	yipqrpymsvgslrdqviypdsvedmrrkgyseqqletildivhlhhilqreggwealcd	600
Opposum	yipqrpymsvgslrdqviypdtvedmkrkgysdsyleaildivnlnhivqreggweascd	521
Orca	yvpqrpymsvgslrdqviypdsvedmrrkgfseqhleaildivhlhhilqreggweavcd	600
Prairie-vole	yipqrpymsvgslrdqviypdsaedmrrkgcseqqleailaivhlrhilqreggweavcd	600
Python	yipqrpymsvgtlrdqviypdtfkdmrrkghtdsdlerildivnlnyivqreggweavsd	541
Small-eared-galago	yipqrpymsvgslrdqviypdsvedmrrkgyserhleaildivhlhhilqreggweamcd	600
Sperm-whale	yvpqrpymsvgslrdqviypdsvedmrrkgfseqhleaildivhlnhilqreggweavcd	600

Human	wkdvlsggekqrigmarmfyhrpkyalldectsavsidvegkifqaakdagiallsithr	660
Chimpanzee	wkdvlsggekqrigmarmfyhrpkyalldectsavsidvegkifqaakdagiallsithr	660
Elephant	wkdvlsggekqrigmarmfyhrpkyalldectsavsidvegkifqaakdagiallsithr	660
Gorilla	wkdvlpggekqrigmarmfyhrpkyalldectsavsidvegkifqaakdagiallsithr	660
Green-Monkey	wkdvlsggekqrigmarmfyhrpkyalldectsavsidvegkifqaakdagiallsithr	656
Jerboa	wkdvlsggekqrigmarmfyhrpkyalldectsavsidvegkifqaakdagiallsithr	660
Macaque	wkdvlsggekqrigmarmfyhrpkyalldectsavsidvegkifqaakdagiallsithr	660
Marmoset	wkdvlsggekqrigmarmfyhrpkyalldectsavsidvegkifqaakdtgiallsithr	660
Mouse	wkdvlsggekqrigmarmfyhrpkyalldectsavsidvegkifqaakdagiallsithr	660
Orangutan	wkdvlsggekqrigmarmfyhrpkyalldectsavsidvegkifqaakdagiallsithr	660
Panda	wkdvlsggekqrigmarmfyhrpkyalldectsavsidvegkifqaakdagiallsithr	660
Pig	wkdvlsggekqrvgmarmfyhrpkyalldectsavsidvegkifqaakdagiallsithr	660
Pika	wkdvlsggekqrigmarmfyhrpkyalldectsavsidvegkifqaakdagiallsithr	660
Prairie-deer-mouse	wkdvlsggekqrigmarmfyhrpkyalldectsavsidvegkifqaakdagiallsithr	660
Rabbit	wkdvlsggekqrigmarmfyhkpkyalldectsavsidvegkifqaakdagiallsithr	660
Rat	wkdvlsggekqrigmarmfyhrpkyalldectsavsidvegkifqaakdagisllsithr	660
Rhesus-Monkey	wkdvlsggekqrigmarmfyhrpkyalldectsavsidvegkifqaakdagiallsithr	660
Seal	wkdvlsggekqrigmarmfyhrpkyalldectsavsidvegkifqaakdagiallsithr	660
Squirrel-Monkey	wkdvlsggekqrigmarmfyhrpkyalldectsavsidvegkifqaakdagiallsithr	660
Tarsier	wkdvlsggekqrigmarmfyhrpkyalldectsavsidvegkifqaakdagiallsithr	660
Tasmanian-devil	wkdvlsggekqrigmarmfyhrpkyalldectsavsidvegkifqaakdagiallsithr	539
Walrus	wkdvlsggekqrigmarmfyhrpkyalldectsavsidvegkifqaakdagiallsithr	660
Water-buffalo	wkdvlsggekqrvgmarmfyhrpkyalldectsavsidvegkifqaakdagiallsithr	597
White-Rhinoceros	wkdvlsggekqrigmarmfyhrpkyalldectsavsidvegkifqaakdagiallsithr	659
Yak	wkdvlsggekqrvgmarmfyhrpkyalldectsavsidvegkifqaakdagiallsithr	642
Aardvark	wkdvlsggekqrvamarmfyhrpkyalldectsavsidvegrifqaakdagiallsithr	654
Alpaca	wkdvlsggekqrvgmarmfyhrpkyalldectsavsidvegkifqaakdagiallsithr	658
Armadillo	wkdvlsggekqrigmarmfyhrpkyalldectsavsidvegkifqaakdagiallsithr	660
Baboon	wkdvlsggekqrigmarmfyhrpkyalldectsavsidvegkifqaakdagiallsithr	660
Bat	wkdvlsggekqrigmarmfyhkpkyalldectsavsidvegkifqaakdagiallsithr	660
Cat	wkdvlsggekqrigmarmfyhrpkyalldectsavsidvegkifqaakdagiallsithr	660
Chinchilla	wkdvlsggekqrigmarmfyhrpkyalldectsavsidvegkifqaakdagiallsithr	660
Chinese-hamster	wkdvlsggekqrigmarmfyhrpkyalldectsavsidvegkifqaakdagiallsithr	660
Cow	wkdvlsggekqrvgmarmfyhrpkyalldectsavsidvegkifqaakdagiallsithr	660
Dog	wkdvlsggekqrigmarmfyhrpkyalldectsavsidvegkifqaakdagiallsithr	660
Dolphin	wkdvlsggekqrvgmarmfyhrpkyalldectsavsidvegkifqaakdagiallsithr	593
Ferret	wkdvlsggekqrigmarmfyhrpkyalldectsavsidvegkifqaakdagiallsithr	660
Florida-manatee	wkdvlsggekqriamarmfyhrpkyalldectsavsidvegkifqaakdagiallsithr	659
Flying-Fox	wkdvlsggekqrigmarmfyhkpkyalldectsavsidvegkifqaakdagiallsithr	660
Golden-hamster	wkdvlsggekqrigmarmfyhrpkyalldectsavsidvegkifqaakdagiallsithr	658
Ground-Squirrel	wkdvlsggekqrigmarmfyhrpkyalldectsavsidvegkifqaakdagiallsithr	658
Guinea-pig	wkdvlsggekqrigmarmfyhrpkyalldectsavsidvegkifqaakdagiallsithr	660
Hedgehog	wkdvlsggekqrigmarmfyhkpkyalldectsavsidvegkifqaakdagiallsithr	659
Horse	wkdvlsggekqrigmarmfyhrpkyalldectsavsidvegkifqaakdagiallsithr	660
Lemur	wkdvlsggekqrigmarmfyhrpkyalldectsavsidvegkifqaakdagiallsithr	660
Minke-whale	wkdilsggekqrvgmarmfyhrpkyalldectsavsidvegkifqaakdagiallsithr	640
Mole-rat	wkdvlsggekqrigmarmfyhrpkyalldectsavsidvegkifqaakdagiallsithr	660
Naked-mole-rat	wkdvlsggekqrigmarmfyhrpkyalldectsavsidvegkifqaakdagiallsithr	660

```
Opposum                  wkdvlsggekqrigmarmfyhrpkyalldectsavsidvegkifqaakdagiallsithr  581
Orca                     wkdvlsggekqrvgmarmfyhrpkyalldectsavsidvegkifqaakdagiallsithr  660
Prairie-vole             wkdvlsggekqrigmarmfyhrpkyalldectsavsidvegkifqaakdagiallsithr  660
Python                   wkdvlsggekqrmgmarmfyhrpkyalldectsavsidvegkifqaakdagiallsithr  601
Small-eared-galago       wkdvlsggekqrigmarmfyhrpkyalldectsavsidvegkifqaakdagiallsithr  660
Sperm-whale              wkdvlsggekqrvgmarmfyhrpkyalldectsavsidvegkifqaakdagiallsithr  660


Human                    pslwkyhthllqfdgeggwkfekldsaarlslteekqrleqqlagipkmqrrlqelcqil  720
Chimpanzee               pslwkyhthllqfdgeggwkfekldsaarlslteekqrleqqlagipkmqrrlqelcqil  720
Elephant                 pslwkyhthllqfdgeggwkfekldsaarlslteekqrleqqlagipkmqrrlqelchil  720
Gorilla                  pslwkyhthllqfdgeggwkfekldsaarlslteekqrleqqlagipkmqrrlqelcqil  720
Green-Monkey             pslwkyhthllqfdgeggwkfekldsaarlslteekqrleqqlagipkmqrrlqelcqil  716
Jerboa                   pslwkyhthllqfdgeggwkfekldsaarlslteekqrleqqlagmpemqgrlqelrqil  720
Macaque                  pslwkyhthllqfdgeggwkfekldsaarlslteekqrleqqlagipkmqrrlqelcqil  720
Marmoset                 pslwkyhthllqfdgeggwkfekldsavrlslteekqrleqqlagipkmqrrlqelcqil  720
Mouse                    pslwkyhthllqfdgeggwkfekldsaarlslteekqrleqqlagipkmqgrlqelrqil  720
Orangutan                pslwkyhthllqfdgeggwkfekldpaarlslteekqrleqqlagipkmqrrlqelcqil  720
Panda                    pslwkyhthllqfdgeggwkfekldsaarlslteekqrleqqlagipkmqrrlqelcqil  720
Pig                      pslwkyhthllqfdgeggwkfekldsaarlslteekqrleqqlagipkmqrrlqelcqil  720
Pika                     pslwkyhthllqfdgeggwkfekldsaarlslteekqrleqqlagipkmqrrlqelcqil  720
Prairie-deer-mouse       pslwkyhthllqfdgeggwkfekldsvarlslteekqrleqqlagipkmqgrlqelrqil  720
Rabbit                   pslwkyhthllqfdgeggwkfekldsaarlslteekqrleqqlagipkmqrrlqelcqil  720
Rat                      pslwkyhthllqfdgeggwkfekldsaarlslteekqrleqqlagipkmqgrlqelrqil  720
Rhesus-Monkey            pslwkyhthllqfdgeggwkfekldsaarlslteekqrleqqlagipkmqrrlqelcqil  720
Seal                     pslwkyhthllqfdgeggwkfekldpaarlslteekqrleqqlagipkmqrrlqelcqil  720
Squirrel-Monkey          pslwkyhthllqfdgeggwkfekldsaarlslteekqrleqqlagipkmqrrlqelcqil  720
Tarsier                  pslwkyhthllqfdgeggwkfqkldsaarlslteekqrleqqlagipkmqrrlrelcqil  720
Tasmanian-devil          pslwkyhthllqfdgeggwkfekldtvtrltlreekqrleqqlagipkmqqrlselceil  599
Walrus                   pslwkyhthllqfdgeggwkfekldpaarlslteekqrleqqlagipkmqrrlqelchil  720
Water-buffalo            pslwkyhthllqfdgeggwkfekldsaarlslteekqrleqqlagipkmqrrlqelcqil  657
White-Rhinoceros         pslwkyhthllqfdgeggwkfekldsaarlslteekqrleqqlagvpkmqrrlqelcqil  719
Yak                      pslwkyhthllqfdgeggwkfekldsaarlslteekqrleqqlagipkmqrrlqelcqil  702
Aardvark                 pslwkyhthllqfdgeggwkfqkldsaarlslteekqrleqqlagipkmqrrlqelcqil  714
Alpaca                   pslwkyhthllqfdgeggwkfekldsaarlslteekqrleqqlagipkmqrrlqelcqil  718
Armadillo                pslwkyhthllqfdgeggwkfekldsvarlslteekqrleqqlagipkmqqrlqelcqil  720
Baboon                   pslwkyhthllqfdgeggwkfekldsaarlslteekqrleqqlagipkmqrrlqelcqil  720
Bat                      pslwkyhthllqfdgeggwkfekldsaarlslteekqrleqqlagipkmqqrlqelcqil  720
Cat                      pslwkyhthllqfdgeggwkfekldsaarlslteekqrleqqlagipkmqrrlqelcqil  720
Chinchilla               pslwkyhthllqfdgeggwkfekldsaarlslreerqrleqqlagipkmqqrlqelchil  720
Chinese-hamster          pslwkyhthllqfdgeggwkfekldsavrlslteekqrleqqlagipkmqgrlqelcqil  720
Cow                      pslwkyhthllqfdgeggwkfekldsaarlslteekqrleqqlagipkmqrrlqelcqil  720
Dog                      pslwkyhthllqfdgeggwkfekldsaarlslteekqrleqqlagipkmqrrlqelcqil  720
Dolphin                  pslwkyhthllqfdgeggwkfekldsaarlslteekqrleqqlagipkmqrrlqelceil  653
Ferret                   pslwkyhthllqfdgeggwkfekldsaarlslteekqrleqqlagvpkmqrrlqelcqil  720
Florida-manatee          pslwkyhthllqfdgeggwkfekldsaarlslteekqrleqqlagipkmqrrlrelchil  719
Flying-Fox               pslwkyhthllqfdgeggwkfekldsatrlslteekqrleqqlagipkmqrrlqelcqil  720
Golden-hamster           pslwkyhthllqfdgeggwkfekldsaarlslteekqrleqqlagipkmqgrlqelcqil  718
Ground-Squirrel          pslwkyhthllqfdgeggwkfekldsaarlslteekqrleqqlagipkmqrrlqelrqil  718
Guinea-pig               pslwkyhthllqfdgeggwkfekldsaarlslteerqrleqqlagvpkmqqrleelrhil  720
Hedgehog                 pslwkyhthllqfdgeggwkfekldsaarlslteekqrleqqlagipkmqqrlqelcqil  719
Horse                    pslwkyhthllqfdgeggwkfekldsaarlslteekqrleqqlagipkmqrrlqelcqil  720
Lemur                    pslwkyhthllqfdgeggwkfekldsatrlslteekqrleqqlagipkmqrrlqelrqil  720
Minke-whale              pslwkyhthllqfdgeggwkfekldsaarlslteekqrleqqlagipkmqrrlqelcqil  700
Mole-rat                 pslwkyhthllqfdgeggwkfekldsatrlslteekqrleqqlagipkmqhrlqelrqil  720
Naked-mole-rat           pslwkyhthllqfdgeggwklekldsaarlslteerqrleqqlagvpkmqqrlqelrhil  720
Opposum                  pslwkyhthllqfdgeggwkfekldsatrltlreekqrleqqlagipkmqqrlselceil  641
Orca                     pslwkyhthllqfdgeggwkfekldsaarlslteekqrleqqlagipkmqrrlqelceil  720
Prairie-vole             pslwkyhthllqfdgeggwkfekldsatrlslteekqrleqqlagipkmqgrlqelrqil  720
Python                   pslwkyhthllqfdgeggwrfekldpaarlslreekqrleqqlagvpemqqrlselcqil  661
Small-eared-galago       pslwkyhthllqfdgqggwkfekldsaarlslteekqrleqqlagipkiqqrlqelrqil  720
Sperm-whale              pslwkyhthllqfdgeggwkfekldsaarlslteekqrleqqlagipkmqrrlqelcqil  720


Human                    geavapahvpapspqgpgg-lqgast       745
Chimpanzee               geavapahvpapspqgpgg-lqgast       745
Elephant                 geaaap--------------------       726
Gorilla                  geavapahvpapspqgpgg-lqgast       745
Green-Monkey             geamapaqvpapspqdpgg-lqgast       741
Jerboa                   geaaapgqppeaslpgpgips-----       741
Macaque                  geamapaqvpapspqdpgg-lqgast       745
Marmoset                 geavapaqapaqsp------------       734
Mouse                    geaaapvqplvpgvpt----------       736
Orangutan                geavapahvpapspqgpgg-lqgast       745
Panda                    gqgsgvlqgaapp-------------       733
Pig                      gqgpgvphgaat--------------       732
Pika                     geaaspgqltapslpgpsipt-----       741
```

Prairie-deer-mouse	gevaapvqplvpgvst----------	736
Rabbit	geavtpgqlaapslpgpgvpt-----	741
Rat	geaaapvqplvpgipt----------	736
Rhesus-Monkey	geamapaqvpapspqdpgg-lqgast	745
Seal	gqgsgvlqgaa---------------	731
Squirrel-Monkey	geavapaeapapspqgpgg-lqgast	745
Tarsier	geavapgqspapglqgpsvapgast-	745
Tasmanian-devil	gedadlhhvsqtsrpsp---------	616
Walrus	gqgsgvlqgaapp-------------	733
Water-buffalo	gqgpganqgaat--------------	669
White-Rhinoceros	gqglqgatt-----------------	728
Yak	gqgpganqgtat--------------	714
Aardvark	gesavpgqp-----------------	723
Alpaca	gqgpsglqgaat--------------	730
Armadillo	geaaapqqplapevat----------	736
Baboon	geamapaqvpapspqdpgg-lqgast	745
Bat	gqgrrvvqgaat--------------	732
Cat	gqgsgvlqgaasp-------------	733
Chinchilla	getpgpavpp----------------	730
Chinese-hamster	getaapvqplvpgvpt----------	736
Cow	gqgpganqgtat--------------	732
Dog	gqgsgvlqgavpp-------------	733
Dolphin	gqgpgvlqaaat--------------	665
Ferret	gqgpgvlqgaplp-------------	733
Florida-manatee	geeaapeqppapsppgpglsrgatt-	744
Flying-Fox	gqgpkvlqgadi--------------	732
Golden-hamster	getaapvkplvpdvpt----------	734
Ground-Squirrel	geamapqqppaaslpgpgvpt-----	739
Guinea-pig	geatgpgips----------------	730
Hedgehog	geaatsgsppassppgpgllpgitt-	744
Horse	gqglqgatt-----------------	729
Lemur	geavapgqppsvl-qgtat-------	738
Minke-whale	gqgpgvlqaaat--------------	712
Mole-rat	geaaap-qpmaaslpgpgvpt-----	740
Naked-mole-rat	geap----------------------	724
Opposum	aedadsrpvpqtsdp-----------	656
Orca	gqgpgvlqaaat--------------	732
Prairie-vole	geaaapvqplvpgvpt----------	736
Python	gedapsamg-----------------	670
Small-eared-galago	geavapgqppvlrpqgpgsglqgass	746
Sperm-whale	gqgpgvlqaaat--------------	732
